# A Minimally Invasive Surgical Procedure to Harvest Palate Periosteum as a Source of Mesenchymal Stromal/Stem Cells for Bone Tissue Engineering

**DOI:** 10.3390/dj12060172

**Published:** 2024-06-05

**Authors:** André Antonio Pelegrine, David Gonzalo Montero López, Antonio Carlos Aloise, João Pedro Grandini Zeferino, Carolina Guassi Mannina, Raul Canal, Daniel Navarro da Rocha, Tamara Cristina Lopes de Castro, Elizabeth Ferreira Martinez, Lexie Shannon Holliday, Roberto Dalto Fanganiello, José Ricardo Muniz Ferreira

**Affiliations:** 1 Division of Implant Dentistry, Faculdade São Leopoldo Mandic, Campinas 13045-755, Brazil; gmontero@uce.edu.ec (D.G.M.L.); aloiseac@gmail.com (A.C.A.); jpedro.zeferino@gmail.com (J.P.G.Z.); carolmannina5@gmail.com (C.G.M.); 2 ANADEM, Brasília 70322-901, Brazil; presidencia@anadem.org.br; 3 Department of Bioengineering, R-Crio Criogenia S.A., Campinas 13098-324, Brazil; navarro@r-crio.com (D.N.d.R.); tamara@r-crio.com (T.C.L.d.C.); josericardo@r-crio.com (J.R.M.F.); 4 Division of Oral Pathology and Cell Biology, Faculdade São Leopoldo Mandic, Campinas 13045-755, Brazil; elizabeth.martinez@slmandic.edu.br; 5 Department of Orthodontics, University of Florida, Gainesville, FL 32611, USA; sholliday@dental.ufl.edu; 6 Faculty of Science and Engineering, Université Laval, Quebec City, QC G1V 0A6, Canada; robertofanganiello@gmail.com

**Keywords:** periosteum, cell differentiation, mesenchymal stem cells, periosteum-derived cells, hard palate, minimally invasive surgical procedures

## Abstract

The aim of this study is to validate a minimally invasive surgical procedure to harvest palate periosteum as a source of tissue for mesenchymal stromal/stem cells. We performed a standardized procedure to harvest the palate periosteum in ten subjects, which consisted of a 3 mm disposable punch and a Molt periosteal elevator to harvest a small full-thickness fragment of soft tissue at the hard palate area, between the upper bicuspids, 3 to 4 mm apical to the cement enamel junction. The one-third inner portion was fragmented, and following standard cell culture procedures, the adherent cells were cultured for three passages, after obtaining 70–90% confluence. Cell morphology analysis, flow cytometry analysis, and viability and osteogenic differentiation assays were performed. In all 10 cases, uneventful healing was observed, with no need for analgesic intake. The evaluation of cell morphology showed elongated spindle-shaped cells distributed in woven patterns. A high viability range was verified as well as an immunophenotype compatible with mesenchymal stem cell lineage. The differentiation assay showed the potential of the cells to differentiate into the osteogenic lineage. These results demonstrate that the minimally invasive proposed surgical technique is capable of supplying enough periosteum source tissue for stem cell culture and bone tissue engineering.

## 1. Introduction

Mesenchymal stem cells (MSCs) are cells capable of self-renewal and multilineage differentiation [1]. MSCs are present in a variety of tissues and can be isolated at any stage of an individual’s life, maintaining their ability to adhere and multiply in vitro. After being separated from the dental pulp and characterized by Gronthos et al. (2002) [2], the MSCs identified in the cranio-orofacial region gained enormous significance. This cell type has been suggested since then to be used in several experimental and clinical protocols for regenerative medicine. Besides that, several other oral cavity source tissues for obtaining MSCs were studied, including periodontal ligament [3], apical papilla [4], dental follicle [5], gingival tissue [6], dental bud [7], periapical cyst [8], and periosteum [9].

The periosteum is a tissue that covers the external surface of bones and serves as a site of transition between the cortical bone and the surrounding soft tissues and muscles [10]. This tissue contributes to bone regeneration, which has been suggested to be greater than marrow cells [11]. Moreover, the periosteum is primarily responsible for bone callus formation [12]. Histologically, it is divided into two layers, an external (superficial) layer that is more fibrous and an inner (internal) layer that is highly cellular and directly contacts bone. Due to its high vascularity, the periosteum has a large number of endothelial pericytes [13]. According to Praskunas et al. (2021) [14], proteins released by periosteum-derived stem cells (PDSCs) can stimulate osteogenesis, neurogenesis, and angiogenesis, in addition to having immunological repercussions.

PDSCs have been obtained from different sites, such as the knee [15], the mastoid bone [16], the tibia [17], the femur [18], and different oral sites [19]. PDSCs obtained from these oral sites have shown a high potential of self-commitment to the osteoblast lineage in culture and have been traditionally obtained during teeth extraction [19], especially in procedures that demand a surgical flap design, such as in palatally embedded canines [9]. PDSCs have also been obtained from oral sites during palatal repair surgeries [20]. Even in situations where no previous surgical treatment is required, the oral periosteum is also usually harvested from a surgical flap designed just for harvesting purposes [21]. Wang et al. (2018) [21] carried out a huge palatal flap surgery, with a deep vertical releasing incision, to harvest periosteum tissue for stem cell obtaining. This is clearly a drawback of this harvesting method due to the presence of the greater palatine neurovascular bundle in the palate, which might result in a hemorrhage if injured [22].

As there are examples of tissue sources with small volumes obtained from oral sites, such as dental pulp, one may argue if a periosteum flap-designed surgery is really necessary to isolate stem cells for bone tissue engineering. If so, evidently a minimally invasive procedure should be preferred, as an intra-oral surgical flap is commonly related to bleeding, pain, and swelling [23]. To the best of our knowledge, there is no scientific publication that studied the use of PDSCs obtained through a flapless procedure. Therefore, the aim of this study was to verify if a small (3 mm) punch of soft tissue from the human palate is sufficient for MSC isolation, culture, and osteogenic differentiation.

## 2. Materials and Methods

### 2.1. Selection of Subjects

The inclusion criteria included systemically healthy patients of both genders, aged 18 and above. Patients were excluded if they had a history of soft tissue grafts removed from the palatal area. The study design was approved by the Research Ethics Committee of Faculdade São Leopoldo Mandic (number 6.765.029; CAAE: 76220223.0.0000.5374), and free and informed consent forms for all patients were obtained in accordance with the Declaration of Helsinki. The study was conducted in compliance with Good Cell Culture Practices [24].

### 2.2. Surgical Procedure and Pre- and Postoperative Care

**Figure 1:**
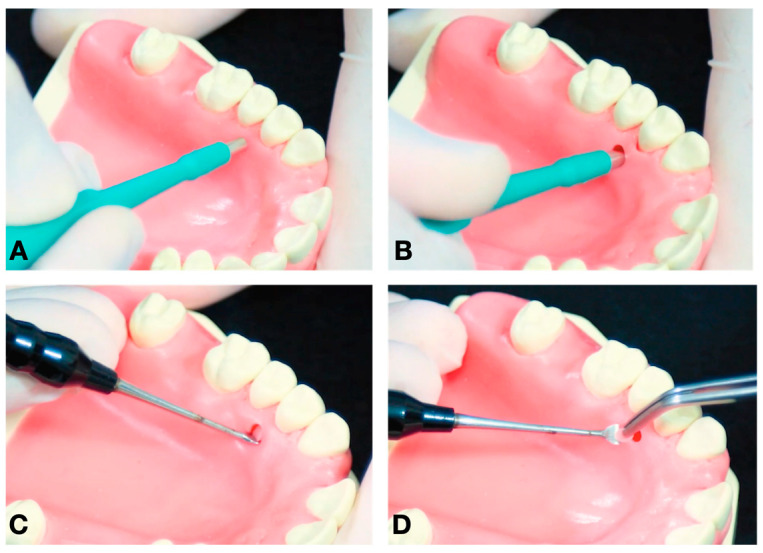
Stages of the surgical procedure: (**A**) a disposable 3 mm punch was inserted in the hard palate area between the upper bicuspids, 3 to 4 mm apical to the cement enamel junction; (**B**) a rotational movement during the punch incision was performed, both clockwise and counterclockwise. It was also necessary to apply pressure on the scalpel until it touched the bone tissue, aiming to remove the periosteum layer; (**C**) a Molt periosteal elevator was used to harvest full-thickness gingival tissue from the hard palate area; (**D**) in the end, tweezers were used for the adequate insertion of the collected tissue into the tube containing the transportation medium.

**Figure 2:**
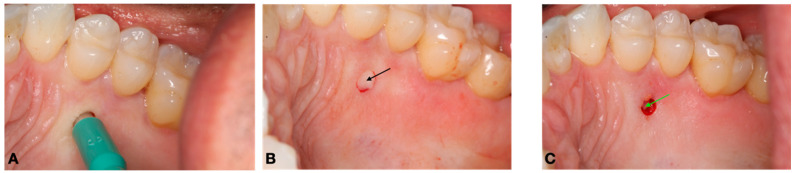
Clinical Procedure: (**A**) incision with the disposable punch; (**B**) incised hard palate tissue (black arrow); (**C**) donor site after the tissue removal. Note the small exposure of the cortical bone (green arrow).

**Figure 3:**
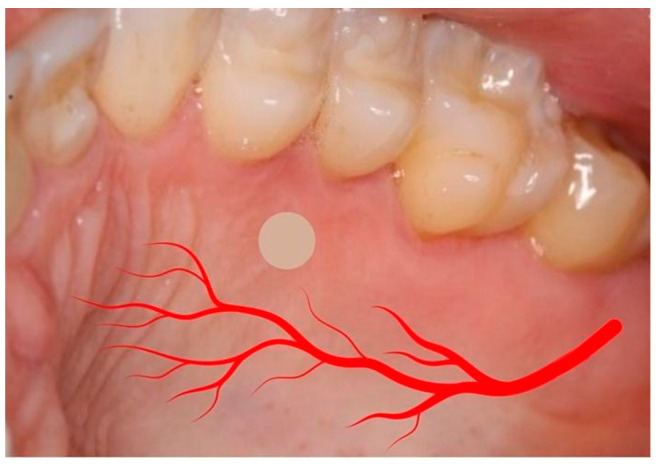
Site for tissue collection. Considering the hard palate anatomy and vascular topography, the safest donor site is located between the upper bicuspids, near 3 to 4 mm apical to the cement enamel junction. Note the simulation of the greater palatine neurovascular bundle in red.

**Figure 4:**
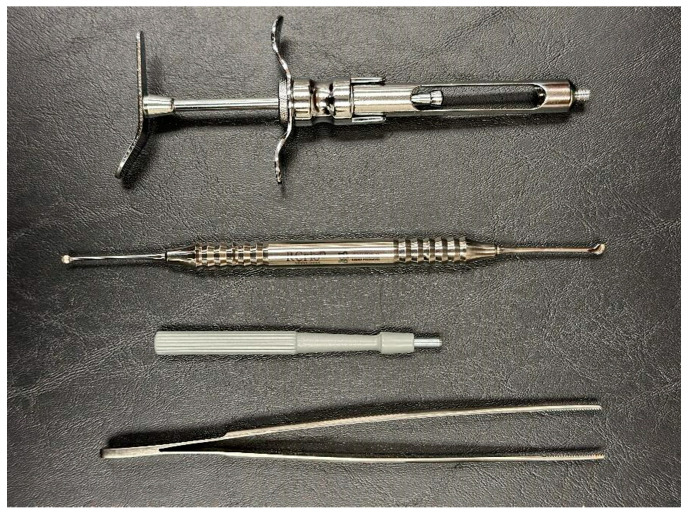
Instrumentals used for palate periosteum harvesting.

Chlorhexidine 0.12% and 2% were used for intra- and extra-oral antisepsis, respectively. After the administration of local anesthesia (2% lidocaine with adrenaline 1:100,000), a disposable 3 mm punch (Disposable Biopsy Punch, Kai Medical, Japan) and a Molt periosteal elevator were used to harvest a full-thickness gingival tissue from the hard palate area (Figure 1 and Figure 2), between the upper premolars, 3 to 4 mm apical to the cement enamel junction to prevent hemorrhage, based in the study of Reiser et al. (1996) (Figure 3). With the aid of tweezers, the collected tissue was transferred into a tube containing the transport medium, which contained Dulbecco’s modified Eagle medium (DMEM, Gibco, Billings, MT, USA) and 1% antibiotic–antimycotic solution (penicillin–streptomycin) (Sigma, St. Louis, MO, USA). A sterile gauze pad was used for homeostasis for 5 min, with no suture required. The instrumentals used in the surgical procedure are shown in Figure 4.

Postoperative instructions included the use of 0.12% chlorhexidine mouthwash and, in case of pain, analgesics (ibuprofen 400 mg, 8/8 h).

### 2.3. Tissue Processing and Cell Culture

Immediately after tissue harvesting, with the aid of a tweezer, the specimen was delicately placed into a Falcon tube containing Dulbecco’s modified Eagle medium (DMEM, Gibco, Billings, MT, USA) and 1% antibiotic–antimycotic solution (penicillin–streptomycin) (Sigma, St. Louis, MO, USA) to deliver the tissue to the laboratory, which occurred in less than 24 h. The tissue was meticulously washed in a laminar flow chamber to ensure its sterility and optimal condition for subsequent processing. The tissue was first rinsed for 1 min in a solution comprising phosphate-buffered saline (PBS) containing 1% penicillin–streptomycin–amphotericin (Sigma, St. Louis, MO, USA). Subsequently, a 1 min wash with a PBS solution containing Mycozap (1:500 mL) was performed (Capsugel, Sidney, Australia). For the final wash, lasting 1 min, a PBS solution containing penicillin–streptomycin (Sigma, St. Louis, MO, USA) was used.

**Figure 5:**
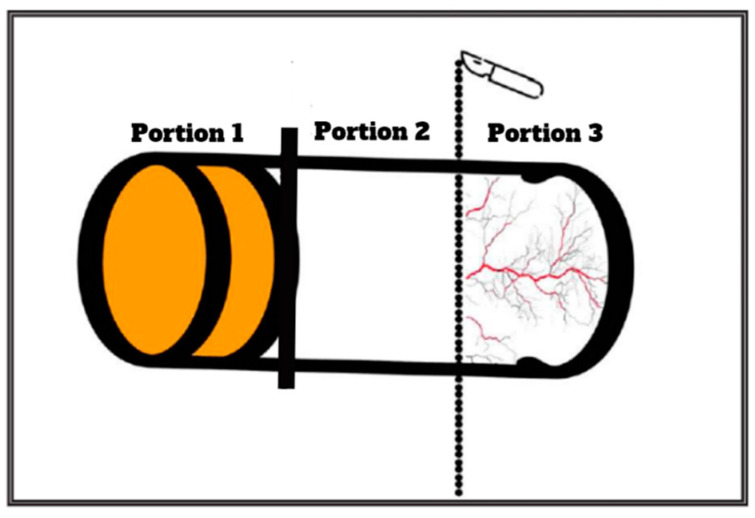
Schematic illustration detailing the collection of periosteal tissue from the specimen, highlighting the presence of epithelium (portion 1), connective tissue (portion 2), and periosteum (portion 3). An incision was performed between portions 2 and 3, with only portion 3 being employed for the isolation of mesenchymal stem cells (MSCs).

**Figure 6:**
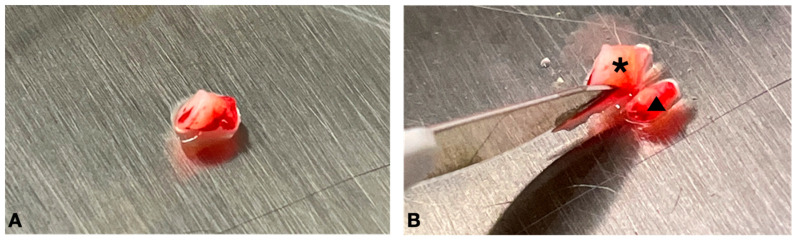
(**A**) Photographic depiction of the collected tissue; (**B**) surgical dissection of the tissue delineating portion 2 from portion 3. The asterisk (*) denotes the 2/3 superficial portion of the tissue, comprising the epithelium and connective tissue, which were subsequently discarded. The remaining 1/3 inner portion (indicated by the arrowhead) containing the periosteum was employed for the isolation and subsequent cultivation of mesenchymal stem cells (MSCs).

The tissue specimen, comprising distinct layers (i.e., epithelium, connective tissue, and periosteum), was further processed. A precise incision was made using a surgical blade to separate the connective tissue from the periosteum. The 2/3 superficial portion of the tissue (comprising the epithelium and connective tissue) was discarded, and the remaining 1/3 inner portion, which contained the periosteum was used for MSC isolation (see Figure 5 and Figure 6).

Subsequently, the 1/3 inner portion containing the periosteum was fragmented with the aid of a 15C blade scalpel. The fragmented tissue was then subjected to enzymatic digestion with a collagenase solution at a concentration of 6 mg/mL for a duration of 30 min. Once the digestion process was completed, the enzymatic activity was promptly neutralized, and the resultant mixture was subjected to centrifugation at 300× *g* for 5 min. The resulting pellet, containing the isolated cells, was carefully collected and subsequently placed into 25 cm^2^ plastic culture flasks with a xeno-free medium (code H4522, Sigma-Aldrich, St. Louis, MO, USA).

Adherent cells were cultured up to three passages, and cell culture was continued until they reached a confluence level of 70–90%. Finally, the harvested MSCs were cryopreserved to ensure their long-term availability for future research.

### 2.4. Adhesion, Morphology, and Viability Assays

For cell adhesion assay, the adherent viable cells were counted after trypsinization (TrypLE™ Express Stable Trypsin Replacement Enzyme without Phenol Red, Life Technologies Corporation, Roskilde, Denmark) using the trypan blue exclusion approach and a Neubauer hemocytometer. Morphological analyses were performed using phase-contrast microscopy, after 3 days.

### 2.5. Immunophenotyping

Stem cells from the third passage were used for immunophenotypic characterization. Cells were trypsinized, and the cellular suspension was centrifuged at 300× *g* for 5 min. Cells were stained with antibodies conjugated with fluorescein isothiocyanate (FITC) or phycoerythrin (PE) to determine the expression of CD-105-PE, CD-73-PE, CD-90-FITC, and CD-45-FITC (BD Pharmingen, San Diego, CA, USA). Cells were resuspended in 0.1 mL stain buffer BSA (BD Pharmingen, San Diego, CA, USA) and incubated with FITC- or PE-conjugated antibodies for 30 min at room temperature and protected from light. The samples were analyzed by flow cytometry to identify specific fluorescence channels of each antibody.

### 2.6. Osteogenic Differentiation

Mineralized matrix formation was analyzed by alizarin red staining (ARS). The cells were plated at an initial cell density of 2 × 10^4^ cells/well in 24-well culture plates and cultured in 1ml of DMEM high-glucose medium (Gibco, Grand Island, USA), enriched with 10% fetal bovine serum (Gibco, Grand Island, NE, USA), 100 U/mL penicillin and 100 μg/mL streptomycin (Gibco, Grand Island, NE, USA), 5 μg/mL ascorbic acid (Sigma-Aldrich, St. Louis, MO, USA), 7 mM β-glycerophosphate (Sigma-Aldrich, St. Louis, MO, USA), and 10-7M dexamethasone (Sigma-Aldrich, St. Louis, MO, USA). The culture medium was replaced every 2–3 days. The cells were cultured in an incubator at 37 °C in a humidified atmosphere containing 5% CO_2_ and 95% air for up to 21 days.

To confirm osteoblast differentiation, alizarin red staining was used for the detection of mineralized extracellular matrix. Briefly, the cells were fixed using 10% formalin for 30 min, dehydrated in increasing concentrations of ethanol, and incubated with alizarin red staining (Sigma-Aldrich, St. Louis, MO, USA) at room temperature for 10 min. After drying, digital photomicrographs were obtained from representative areas from each slide using an Olympus IX70 microscope equipped with an AmScope digital camera.

### 2.7. Analgesic Intake

All patients were asked to record the quantity of analgesic (ibuprofen 400 mg) intake during the first 7 days after surgery.

## 3. Results

Eight male and two female subjects were enrolled in this study, with a mean + standard deviation age of 49.30 ± 16.03 years. The demographic data of all subjects are shown in Table 1.

### 3.1. Adhesion and Morphology

**Figure 7:**
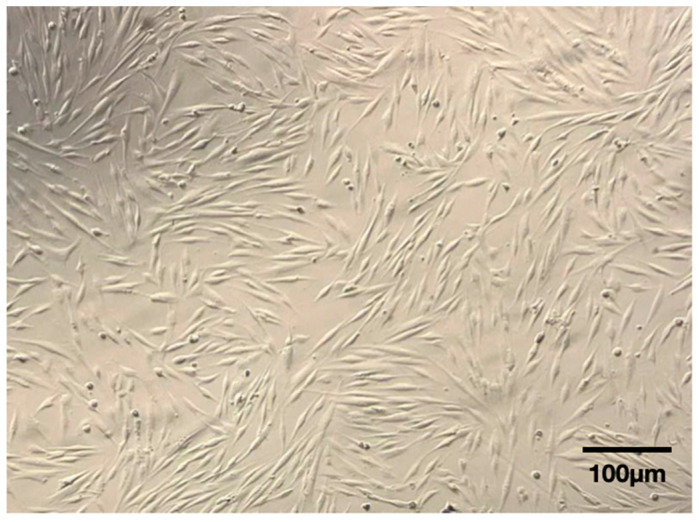
The cells were spindle-shaped and attached to the cell culture flask after 3 days of the primary culture (100×).

The cells were able to adhere to the plastic surface and exhibited an elongated spindle-shaped morphology (Figure 7).

### 3.2. Viability Assay and Immunophenotyping

**Figure 8:**
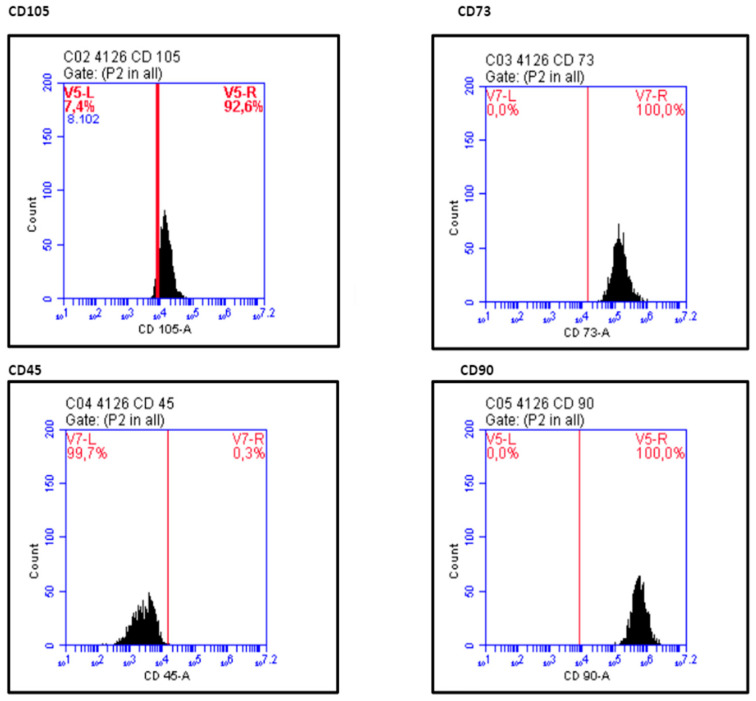
CD105, CD73, CD90, and CD45 marker expression profiles.

The mean ± standard deviation viability percentage was 94.00 ± 3.84%. Flow cytometric assay showed the following pattern: CD105+, CD73+, CD90+, and CD45− (Figure 8). Table 2 shows the results for the 10 patients.

### 3.3. Osteogenic Differentiation

**Figure 9:**
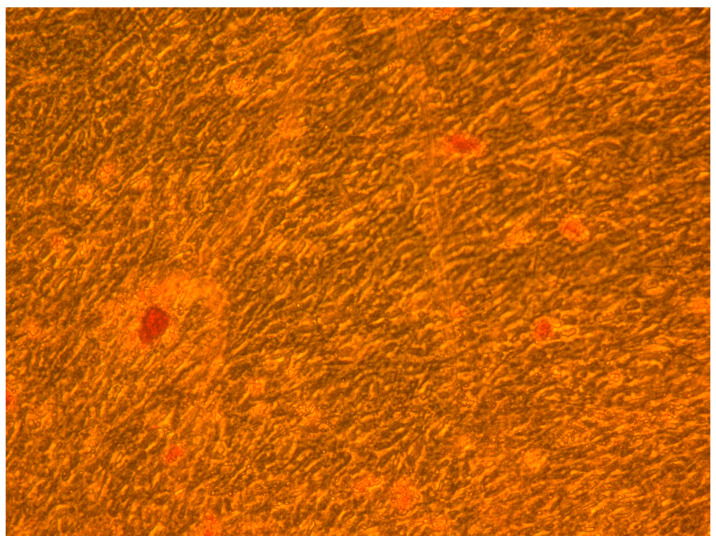
Mineralized extracellular matrix detected by alizarin red staining. Original magnification: 40×.

**Figure 10:**
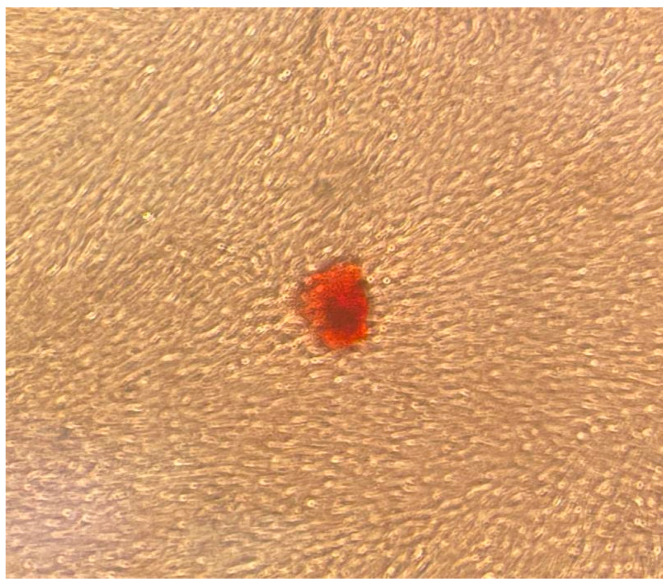
Mineralized extracellular matrix detected by alizarin red staining. Original magnification: 100×.

A mineralized extracellular matrix was detected by alizarin red staining (Figure 9 and Figure 10).

### 3.4. Analgesic Intake

All 10 patients showed uneventful healing, with no need for analgesic intake.

## 4. Discussion

It is current knowledge that the source tissue of stromal/stem cells must be selected according to the purpose of the desired therapy. Studies comparing the performance of mesenchymal stem cells derived from bone marrow and adipose tissue showed that bone marrow is a better tissue source for bone regeneration [25,26]. On the other hand, for soft tissue reconstruction, the opposite was shown [27], and it was suggested that the epigenetic memory obtained from either bone marrow or adipose tissue favors MSC differentiation along an osteoblastic or adipocytic lineage [26]. Furthermore, it seems that even using the same source tissue but harvested from different places would result in different potentials. In this sense, Groeneveldt et al. (2020) [28] showed that alkaline phosphatase and osteocalcin displayed higher mRNA expression in in vitro cultured human PDCs obtained from the maxilla compared to the tibia. This could underlie the higher in vitro osteogenic potential of maxilla-derived PDCs.

PDSCs were correlated with a higher potential for both bone and chondrogenic differentiation, when compared with bone marrow and adipose tissue, respectively [29]. In this scope, a recent systematic review showed the same in vivo bone-forming efficacy between bone marrow and periosteum-derived mesenchymal stem cells [30]. As bone marrow harvesting is a more invasive and difficult strategy than intra-oral periosteum harvesting [19], it is important to study the periosteum as a tissue source for bone tissue engineering.

The fibroblastic morphology of the cells cultured in the present study was compatible with MSCs (i.e., elongated spindle-shaped cells distributed in woven patterns). Moreover, the cells could attach to plastic and were positive for the surface antigens CD 105, CD 73, and CD 90 (89.29 ± 9.51%, 99.83 ± 0.43%, and 99.48 ± 1.26%, respectively) and negative for the hematopoietic antigen CD 45 (8.96 ± 1.74%). In summary, these data indicate that these cells are positive for mesenchymal cell line markers and negative for hematopoietic markers. The cells were also able to differentiate into the osteoblastic lineage. These criteria are considered useful for determining mesenchymal stem cells [31]. The immunophenotyping findings presented in our study seem to be even more favorable than those obtained by Trovato et al. (2015) [32], who also worked with the periosteum derived from the palate and obtained positive results for CD 105, CD 73, and CD 90 (52%, 82%, and 82%, respectively) and negative results for CD 45. Caballero et al. (2010) [20] and Wang et al. (2018) [21], who also worked with palate periosteum stem cells, showed lower levels of CD 90 (48% and 75.6%, respectively), when compared with the present study results. As CD 90 expression decreases with differentiation (Caballero et al. (2010)) [20], the higher CD 90 expression of the present study in relation to previously published data might be related to a more prone capability of osteoblastogenic lineage by using the presented protocol. However, this capability should be tested by future in vivo studies.

In the present study, all samples yielded MSC colonies, with a successful stem cell isolation rate of 100% (10 within 10 subjects). This result is more favorable than those obtained by Wang et al. (2018) [21], who harvested the palate periosteum using a surgical flap approach and reached a 58.8% rate (20 within 34 subjects). The 94.00 ± 3.84% viability percentage of PDSCs obtained in the present study is compatible with the >90% rate found in the current literature for adipose stem cells (Kim et al. (2020)) [33], iliac crest bone marrow stem cells (Narbona-Carceles et al. (2014)) [34], and dental pulp stem cells (Vendramini et al. (2021)) [35], and superior to the 73% periosteum stem cell viability reported by Trovato et al. (2015) [32].

The periosteum has osteogenic potential, is a critical element of the bone-healing complex, and is considered the major factor responsible for bone callus formation [36]. MSCs from the periosteum may be primed for osteogenesis and, therefore, an adequate source for bone tissue engineering [16]. Intra-oral periosteum harvesting for MSC isolation and culture is traditionally obtained through surgical flap procedures [9,19,20,21]. As surgical flap procedures have been related to higher levels of pain intensity and associated analgesic consumption when compared with flapless surgery [37], it is important to study the possibility of a flapless approach to obtain PDSCs. Furthermore, the design of deep incisions and a huge flap at the posterior palate can result in hemorrhage [22].

The surgical approach for soft tissue harvesting in the palate is extensively discussed in the periodontology field, as soft tissue defects are very common in the adult population [38]. In this sense, Reiser et al. (1996) [39] drew clinicians’ attention to the fact that variations in the size and shape of the hard palate affect the location of the greater palatine neurovascular bundle. According to the authors, the neurovascular bundle may be located 7 to 17 mm from the cementoenamel junctions of the maxillary premolars. In deep, average, and shallow palatal vaults, the average distance from cementoenamel junctions of the maxillary premolars to the neurovascular bundle is 7 mm, 12 mm, and 17 mm, respectively. In the anterior palatal region (i.e., canines and incisors), the artery drops inferiorly, decreasing the distance from the artery to the cementoenamel junctions of the anterior teeth, and the same can be stated for the posterior teeth (especially the second and third molars). Therefore, the safer site to collect soft tissue while avoiding the occurrence of bleeding, paresthesia, and anesthesia is the premolar area. According to Fu et al. (2011) [40], the distance between the cementoenamel junction to the neurovascular bundle of the premolar site is 12.2 ± 2 mm. These authors stated that this distance is 4 mm less in cases of shallow palatal vaults. Therefore, as our protocol involves a distance of 3 to 4 mm apical to the cement enamel junction of the premolars for the punch incision, a safe zone is still maintained in relation to the neurovascular bundle as well as the premolars’ gingival margin.

The implant dentistry literature shows that flapless surgeries result in less pain and less medicine intake when compared with the standard implant placement after a flap surgery [41]. These data were corroborated by a recent systematic review with meta-analysis, which concluded that flapless surgeries for implant placement are less invasive than the standard flap surgical procedure [42]. In this sense, the minimally invasive protocol for the palate periosteum harvesting used in this study resulted in no need for analgesic intake, which may be related to the lack of a surgical flap design with our protocol. This can lead to a more appropriate use of cell therapy in the dentistry field, possibly clinically improving the bone tissue engineering armamentarium as an alternative for autologous bone grafts.

## 5. Conclusions

The results of the present study allow us to conclude that the proposed flapless surgical technique is minimally invasive and capable of supplying enough periosteum tissue for stem cell isolation and culture to be used in bone tissue engineering.

## 6. Patents

Patent # BR 10 2021 003512 9 is related to the work reported in this manuscript.

## Figures and Tables

**Table 1:** Demographic data.

NUMBER	AGE	GENDER
1	51	M
2	43	M
3	55	M
4	18	F
5	77	M
6	44	M
7	36	M
8	52	M
9	66	F
10	51	M
Mean	49.30	-
Standard Deviation	16.03	-

**Table 2:** Analysis of viability and immunophenotyping.

Patient Number	% Viability	% CD 105	% CD 73	% CD 90	% CD 45
1	95	68.5	100	99.8	8.6
2	87	97.3	100	100	5.9
3	91	98.2	99.9	99.9	40.5
4	100	99.8	100	99.7	0.3
5	95	76.1	98.6	95.9	13.8
6	97	92.6	100	100	0.3
7	91	97	99.9	100	5.3
8	98	92.6	100	100	4.9
9	92	98	99.9	100	4.2
10	94	72.8	100	99.5	5.8
Mean	94	89.29	99.83	99.48	8.96
Standard Deviation	3.84	9.51	0.43	1.26	11.74

## Data Availability

The data presented in this study are available upon request from the corresponding author.
